# Catalase Inhibition by Aminoalkanol Derivatives with Potential Anti-Cancer Activity—In Vitro and In Silico Studies Using Capillary Electrophoresis Method

**DOI:** 10.3390/ijms23137123

**Published:** 2022-06-27

**Authors:** Błażej Grodner, Mariola Napiórkowska, Dariusz Maciej Pisklak

**Affiliations:** 1 Department of Biochemistry and Pharmacogenomics, Medical University of Warsaw, 1 Banacha Street, 02-097 Warsaw, Poland; 2 Department of Biochemistry, Medical University of Warsaw, 1 Banacha Street, 02-097 Warsaw, Poland; mariola.napiorkowska@wum.edu.pl; 3 Department of Physical Chemistry, Medical University of Warsaw, 1 Banacha Street, 02-097 Warsaw, Poland; dpisklak@wum.edu.pl

**Keywords:** catalase inhibitors, aminoalkanol derivatives, anticancer drugs, docking studies

## Abstract

In this work, the investigation of type and inhibitory strength of catalase by two pairs of aminoalkanol derivatives (1,7 diEthyl- and 1,7-diMethyl-8,9-diphenyl-4-azatricyclo (5.2.1.02.6) dec-8-ene- 3,5,10-trione) has been presented. The obtained results allowed for the determination of all kinetic parameters (Km, Vmax, slope angles of Lineweaver–Burk plots, Ki and IC_50_) on the basis of which it was shown that all four aminoalkanol derivatives are competitive inhibitors of catalase. However, the strength of action of each of them depends on the type of substituents present in the main structure of the molecule. Subtle differences in the potency of individual derivatives were possible to detect thanks to the developed, sensitive method of capillary electrophoresis, which allowed simultaneous monitoring of the mutual changes in the concentrations of substrates and products of the reaction catalyzed by the enzyme. Detailed values of kinetic parameters showed that all derivatives are weak inhibitors of catalase, which in this case is a big advantage because each inhibition of catalase activity is associated with a greater amount of accumulated, harmful reactive oxygen species. The results of docking studies also show the convergence of the binding energies values of individual inhibitors with all kinetic parameters of the investigated catalase inhibition and thus additionally confirm the weak inhibitory strength of all four aminoalkanol derivatives.

## 1. Introduction

Catalase (CAT) (EC 1.11.1.6) is a tetrameric enzyme from the oxidoreductase group that catalyzes the process of decomposition of hydrogen peroxide into water and oxygen. It is a very active enzyme and contains a heme moiety at the active site. In mammals, the highest concentration of catalase occurs in the liver and erythrocytes and the lowest in connective tissue. Considerable amounts of catalase are also found in aerobic bacteria and in the peroxisomes of photosynthetic plant cells. Catalase protects cell organelles and tissues from damage by peroxide, which is constantly produced in numerous metabolic reactions [1,2,3,4,5,6,7,8,9,10,11,12,13,14].

Since catalase plays an extremely important role in the processes of deactivation of hydrogen peroxide, which is a factor damaging many cell structures, the regulation of its activity seems to be a very important direction of research. Regulators of catalase activity can be various substances of both natural origin and many synthetic compounds [10,11]. Some of them, such as for example metformin and glucose, are catalase activators [12,13], while others, such as metal ions, flavonoids, 3-Amino-1,2,4-triazole, hydroxylamine, sodium azide, potassium cyanide, 3,3’-diaminobenzidine and its derivatives [14,15,16,17,18], are inhibitors of this enzyme.

The influence of this type of compound on the functioning of catalase in many tissues of various organs may have many consequences related to the proper or incorrect functioning of the whole organism. There are reports of a significant reduction in catalase activity in some serious diseases such as colorectal cancer, gastric adenocarcinoma, H. pylori-infected stomach and Crohn’s disease [19,20,21]. Many other diseases such as cardiovascular diseases, hypertension, Alzheimer’s disease, diabetes mellitus, anemia, vitiligo, some dermatological disorders, schizophrenia and bipolar disorder are also associated with catalase malfunctioning or its deficiency [22,23,24]. Therefore, the administration of substances or consumption of products containing catalase inhibitors may exacerbate the symptoms of the disease and lead to serious consequences. On the other hand, many bacteria, such as H. pylori, Escherichia coli and Salmonella, synthesize catalase (CAT) to defend themselves against the host’s immune response [25,26]. In these cases, research on the use of catalase inhibitors as substances that inhibit the growth and multiplication of bacteria could be an interesting solution, provided that full safety control is maintained.

The introduction of each new substance to treatment is therefore a major challenge. This is what prompted us to conduct a studies to determine the possibilities and number of potential therapeutic effects and side effects caused by the tested compounds. It is well known that enzymes play an extremely important role in maintaining the proper course of biochemical and metabolic processes in our body [27,28]. Disruption of the functioning of one or more enzymes can lead to serious consequences that have a negative impact on the functioning of the entire organism.

Several years ago, a number of aminoalkanol derivatives with potential anti-cancer properties were synthesized and patented [29]. In our previous studies, we have shown that these derivatives (I, II, III, IV), apart from their primary antitumor activity, also inhibited the enzymatic activity of alkaline phosphatase [30], acid phosphatase [31] and acetylcholinesterase [32]. These derivatives were also previously investigated for their determination in biological material [33,34,35,36] and for their cytotoxicity on cancer cell lines such as K562 and HeLa cells [29]. In general, compounds with anti-cancer properties are of great interest in medicine, and their action may additionally affect the activity of many enzymes that protect against cancer. Therefore, in this study we investigated the influence of aminoalkanols (I, II, III and IV) with potential antitumor activity [29] on catalase activity using the capillary electrophoresis (CE) technique.

**Figure 1:**
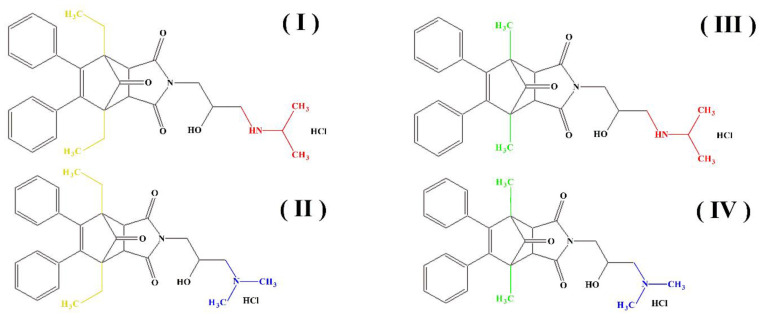
Structures of aminoalkanol derivatives (**I**), (**II**), (**III**) and (**IV**).

These compounds (Figure 1) were evaluated in vitro for their inhibition efficacy of catalase. Toxicity of the studied compounds for chronic myelogenous leukemia cells, K562 and HeLa cells was in the range 400–100 μM [29].

## 2. Results

In this study, the inhibition of enzymatic activity was analyzed in order to determine CAT inhibition by aminoalkanol derivatives.

### 2.1. Experimental

**Figure 2:**
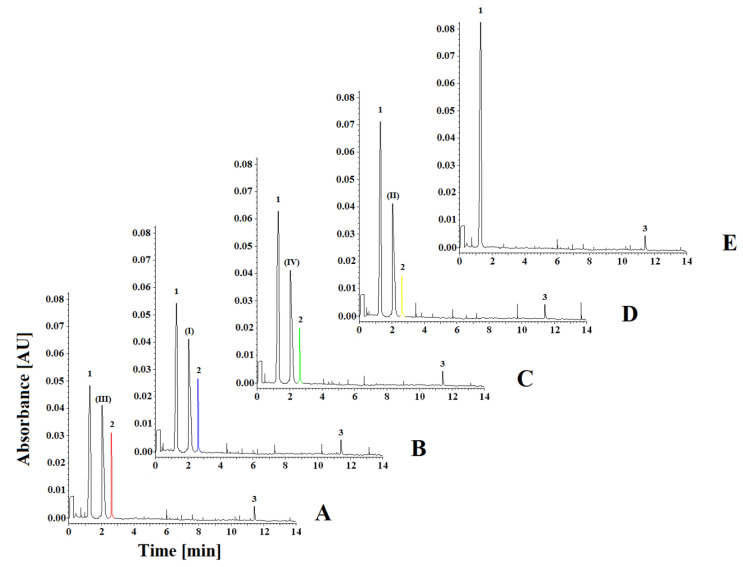
Representative electrophoregrams of investigated compounds in the presence of (I), (II), (III) and (IV) CAT inhibitors at the concentration of 20.00 mM, hydrogen peroxide at the concentration of 10 mM and (1) 20.00 mM of guaiacol (GCOL), (2) tetraguaiacol (TGCOL), (3) catalase (CAT). (**A**): (1) 11.71 mM of GCOL, (2) 8.29 mM of TGCOL (●) in the presence of inhibitor (III). (**B**): (1) 14.14 mM of GCOL, (2) 5.86 mM of TGCOL (●) in the presence of inhibitor (I). (**C**): (1) 15.61 mM of GCOL, (2) 4.39 mM of TGCOL (●) in the presence of inhibitor (IV). (**D**): (1) 17.56 mM of GCOL, (2) 2.44 mM of TGCOL (●) in the presence of inhibitor (II). (**E**): (1) 20.00 mM of GCOL, (2) 0.00 mM of TGCOL without inhibitors.

In this study, we used the capillary electrophoresis (CE) method developed by us to determine the tetraguaiacol complex as a product of the catalase-catalyzed reaction (CAT) (Table 1) in the presence of the enzyme, hydrogen peroxide (HPER) (as a substrate), guaiacol (GCOL) and inhibitors (I), (II), (III) and (IV) (Figure 2). Based on this method, we investigated changes in the concentration of guaiacol reacting with changes in the concentration of hydrogen peroxide depending on the activity of catalase and changes in the concentrations of the formed tetraguaiacol as the final reaction product. The developed CE technique made it possible to measure all the parameters thanks to the possibility of using three wavelengths simultaneously in one analysis. Investigations of kinetic parameters were based on mutual measurements of concentration changes between guaiacol (as the second reactant reacting with hydrogen peroxide) and tetraguaiacol as the final reaction product.

**Figure 3:**
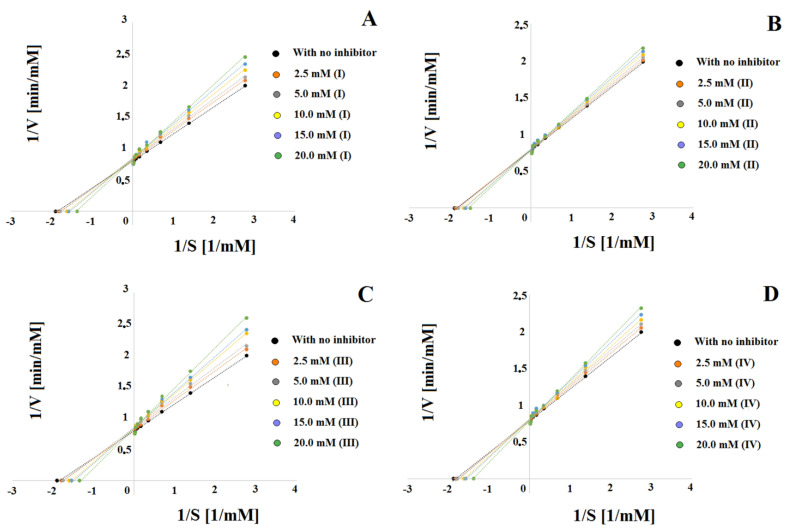
Lineweaver–Burk plots for systems with inhibitors (I) (**A**), (II) (**B**), (III) (**C**) and (IV) (**D**) at concentrations of 2.50, 5.00, 10.00, 15.00 and 20.00 mM.

For the development of this method, we optimized the buffer pH, separating background electrolyte (BGE) concentration, wavelength, temperature, and voltage in accordance with the earlier procedure [32]. The separation of all compounds was investigated at three pH values (7.0, 7.5 and 8.0), three temperatures (20, 25 and 30 °C), four voltages (10, 15, 20 and 25 kV), three concentrations of separating BGE (50, 75 and 100 mM), and four wavelengths (200, 214, 274 and 470 nm). All these separation systems were tested to achieve the best separation parameters, resolution, and analysis time for all components in the reaction mixture. The best separation of tetraguaiacol, guaiacol, catalase (CAT) and compounds (I), (II), (III), (IV) was obtained with 100 mM phosphate buffer (pH 7.5). The measurements were taken at 214, 274 and 470 nm with a fused-silica capillary (effective length: 20 cm, diameter: 50 μm). The capillary temperature was 25 °C, which allowed the best resolution to be achieved. Separation was carried out at 20 kV. Each sample was added to the capillary under hydrodynamic injection. Average detection times for guaiacol (GCOL), compounds (I), (II), (III), (IV), tetraguaiacol (TGCOL) and CAT were 1.25, 2.01, 2.81 and 11.65 min, respectively (Figure 2). The constructed curves were linear over the concentration range of 0.08–10.00 mM used for hydrogen peroxide (HPER) and 0.05–20.00 mM for GCOL and TGCOL. The basic enzymatic activity was investigated using nine concentrations of reaction substrate (HPER) (0.08, 0.16, 0.31, 0.63, 1.25, 2.50, 5.00, 7.50 and 10.00 mM) in the presence of the enzyme (CAT). The effect of inhibition of compounds (I), (II), (III) and (IV) was determined by an enzymatic kinetics study in a system containing successively increasing concentrations of the substrate at a constant concentration (20.00 mM) of inhibitor (I), (II), (III) and (IV). The correlation coefficient for the activity of CAT in the absence of inhibitors was found to be 0.9989, and the slope of the curve was 0.4308. At the concentration of 20.00 mM for compounds (I), (II), (III) and (IV), the correlation coefficients were determined at 0.9949–0.9979, and the curves slopes at 0.8182–0.7918 for compounds (I), (II), (III) and (IV), based on the inhibition type and inhibitory strength (Table 2, Figure 3).

The statistical power for each dataset and each value of the kinetic parameter was achieved by measuring six times each value obtained for the nine concentrations of substrates and reaction products in the system without inhibitors and for four systems in the presence of 20.00 mM inhibitor (I), (II), (III) and (IV). After exceeding the value of 20.00 mM, there was no further increase in inhibition; therefore, the value of 20.00 mM was considered the cutoff value for both inhibitors.

The inhibitory effects of compounds (I), (II), (III) and (IV) on CAT are shown in the form of electrophoregrams in Figure 2.

### 2.2. Analysis

The research was divided into two stages. In the first stage, kinetic studies of catalase were performed in the presence of (I), (II) and (III), (IV) aminoalkanol derivatives in order to determine the type of inhibition of individual derivatives. The second stage was the study of inhibitors strength and the binding strength of individual inhibitors (I), (II) and (III), (IV) to the active center of CAT. Based on the kinetic studies performed, it was found that all four aminoalkanols derivatives showed a competitive type of inhibition (Table 3).

Based on the inhibition data obtained in the steady-state, Lineweaver–Burk plots (Figure 3) were prepared showing the reciprocal relationship of the reaction substrate concentration (hydrogen peroxide) to the reciprocal of the reaction rate.

Since the plots of straight lines with different slopes (Km/Vmax) for the five concentrations of inhibitors (I), (II), (III) and (IV) intersect the y-axis at one point corresponding to the reciprocal of the reaction rate value (Vmax), such a system suggests reversible competitive CAT inhibition. The calculated Km and Vmax values for systems containing different concentrations of compounds (I), (II) and (III), (IV) also indicate a competitive type of inhibition (Table 4).

Based on the obtained kinetic parameters, the inhibition strength and binding strength of the individual inhibitors (I), (II) and (III), (IV) with the active center of CAT were determined by calculating the values of Michaelis–Menten constants (Km), the maximum velocities (Vmax), the inhibition constants (Ki) and half the maximum inhibitory concentration (IC_50_) for five different concentrations of inhibitors (I), (II) and (III), (IV) (Table 4).

Ascendingly changing Km values (from 0.56 to 0.73 mM) for compound (I), (from 0.55 to 0.64 mM) for compound (II), (from 0.57 to 0.77 mM) for compound (III) and (0.56 to 0.71 mM) for compound (IV) and the common, similar Vmax values (ranging from 1.24 to 1.26 mM/min) for the four inhibitors indicate a competitive type of inhibition (Table 4). Based on the differences in the Km values between individual compounds, it can initially be shown that the strongest competitive CAT inhibitor is compound (III) and the weakest one (II). The inhibitory strength for individual inhibitors decreased in the following direction: (III) (Km from 0.57 to 0.77 mM), (I) (Km from 0.57 to 0.77 mM), (IV) (Km from 0.56 to 0.71 mM), (II) (Km from 0.55 to 0.64 mM).

The next value taken into account to determine the inhibitory strength were the slopes of the Lineweaver–Burk straight lines, the values of which increased with the increase of the concentration of inhibitors (I), (II) and (III), (IV) to the concentration limit value of 20.00 mM above which a further increase in inhibition was not observed. Comparing the values of the slope angles for individual inhibitors (I), (II), (III) and (IV) also showed that, in this case, the strongest competitive inhibitor of CAT turned out to be the compound (III) for which the values of the slopes were the highest (from 25.09 to 32.68). The weakest inhibitor was compound (II) and the inhibitory strength for individual inhibitors decreased in the following direction: (III) (25.09 to 32.68), (I) (24.72 to 30.75), (IV) (24.33 to 29.27), (II) (23.70 to 27.06). (Table 4, Figure 3).

To derive the next value determining the inhibitory strength force of CAT by compounds (I), (II), (III) and (IV), the values of the inhibition constants (Ki) were calculated for the five concentrations (2.5, 5.0, 10.0, 15.0, 20.0, 25.0 mM) of these compounds. Moreover, in this case the results clearly indicated that the strongest competitive inhibitor of CAT is compound (III), which showed the strongest binding to the active site of the CAT (Ki from 8.86 to 1.32). The weakest binding to the active site of the CAT was shown by inhibitor (II) (Ki from 47.70 to 3.04) and the strength of the affinity of inhibitors (I), (II), (III) and (IV) to the active site of the CAT decreased in the following direction: (III) (Ki from 8.86 to 1.32), (I) (Ki from 11.73 to 1.61), (IV) (Ki from 14.23 to 1.79), (II) (Ki from 47.70 to 3.04).

**Figure 4:**
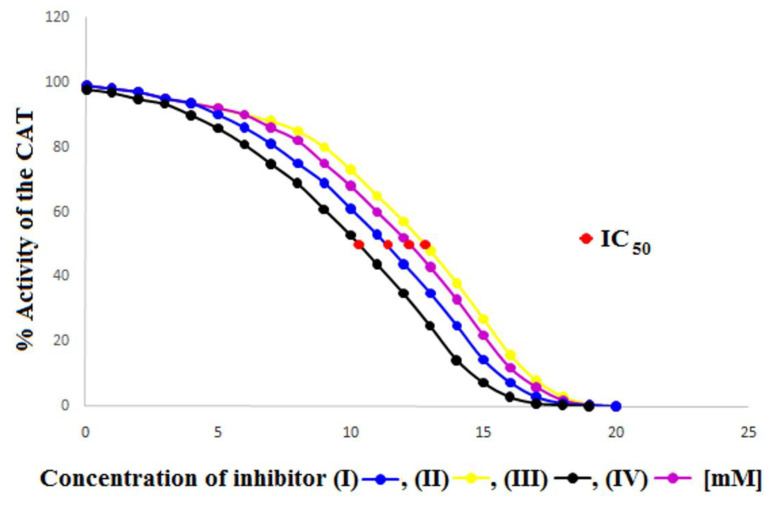
Graphical determination of IC_50_ for (I), (II), (III) and (IV) catalase inhibitors.

In order to obtain information regarding the number of individual compounds (I), (II), (III) and (IV) needed to inhibit CAT by 50% and thus obtain additional values characterizing the potency of compounds (I), (II), (III) and (IV) in inhibiting CAT, the half maximal inhibitory concentration (IC_50_) was calculated (Table 4, Figure 4).

Comparing the obtained IC50 values for compounds (I) (IC_50_ = 11.40 mM), (II) (IC_50_ = 12.80 mM), (III) (IC_50_ = 10.30 mM) and (IV) (IC_50_ = 12.20 mM), it can be clearly seen that compound (III) lowers the CAT activity by 50% at the lowest concentration of all four inhibitors. This means that compound (III) is the most potent CAT inhibitor, which is in line with all previously obtained kinetic parameter values. The IC_50_ values for all four compounds increase in sequence (III) (IC_50_ = 10.30 mM), (I) (IC_50_ = 11.40 mM), (IV) (IC_50_ = 12.20 mM), (II) (IC_50_ = 12.80 mM), again confirming that compound (III) is the most potent CAT inhibitor while compound (II) is the weakest CAT inhibitor.

The research carried out above shows that the inhibitory strength and affinity of all four derivatives depend on the type of four substituents (isopropylamine, dimethylamine, ethyl and methyl) present in the main chemical molecule of compounds (I), (II), (III) and (IV) (Table 5).

Considering the known spatial structure of human catalase and the structure of competing inhibitor molecules, we also analyzed the possibility of their interaction with the enzyme’s active site. The catalytically active form of catalase occurs in the form of a homotetramer with a heme molecule coordinating the iron atom at the active site in each of the subunits. The analysis of the structure of this protein shows that the active site is inside the protein structure with limited access through a narrow long pocket. The preliminary structural analysis suggested that the analyzed compounds, due to their extensive steric structure, would have limited possibilities of penetrating into the pocket and of direct interaction with the active center. To estimate the site of interaction, the potential bioactive conformation of the compounds and possible interactions stabilizing the protein-ligand interaction, molecular docking methods were used. All compounds were docked in the crystallographic structure of human erythrocyte catalase using the Autodock Vina program [37,38]. Docking was performed into the tetramer structure of human erythrocytic catalysis (1DGH). The binding site defined by the size of a 35 × 25 × 25 Å grid in the *x*, *y* and *z* axes, respectively, elongated along the binding pocket, was to take into account both the active site of the enzyme, the interior of the cavity leading to the active site and also the surroundings of the cavity entrance. The resulting predicted binding affinity for all four compounds was similar and ranged from −8.9 and −8.2 kcal/mol. The predicted values of the docking scoring function for all four compounds is presented in Table 5. The higher scoring function (−8.9 kcal/mol) was obtained for compound III and a slightly lower value was predicted for compound I (−8.6 kcal/mol); both (III and I) contained the isopropyl substituent. The prediction values obtained for the compounds containing the dimethyl amino substituent were slightly lower and amounted to −8.4 kcal/mol for compound II and −8.2 kcal/mol for compound IV, respectively. The obtained values were in good agreement with the experimental data.

**Figure 5:**
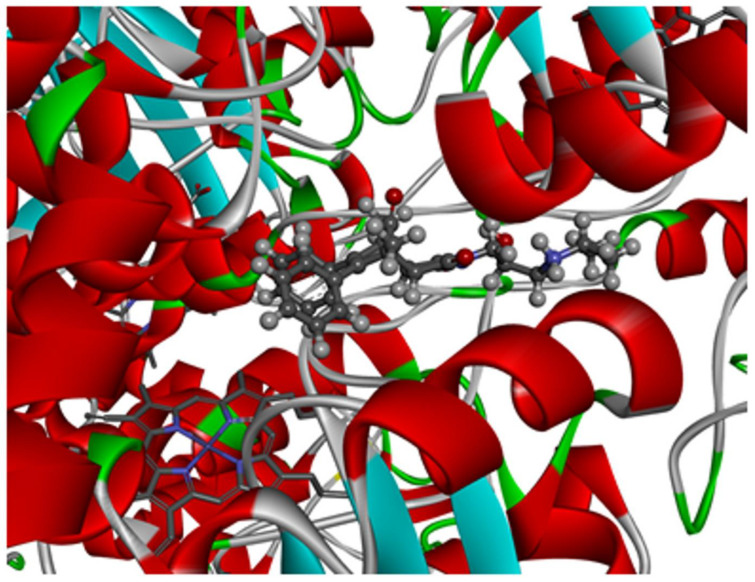
Binding mode of compound III in a pocket of human catalase enzyme.

Another question was how the studied molecules can bind with the active site of hCAT. Analysis of bioactive conformations showed that studied compounds by the presence of spatially expanded imide substituent cannot interact directly with the catalytic site of the enzyme. Through the slight diversity of structures, the bioactive conformations and intramolecular interactions in the ligand-enzyme complex is similar among all the compounds. The obtained binding pose of the highest scored molecule III is depicted in the (Figure 5).

In one of them docking studies showed that they all bind at the entrance to the active site gorge and can present similar binding modes. The dominant interaction stabilizing the complex in all the cases is a hydrogen interaction between the carbonyl groups of the ligands and the side chain of the amino acid SER 254 and the amide atom in the main chain of ALA 123. At the same time, the sterically expanded non-polar fragment of each ligand was stabilized by the interaction of the Pi-alkyl with the aliphatic fragments of the amino acids ALA 117, VAL 126 and ARG127.

**Figure 6:**
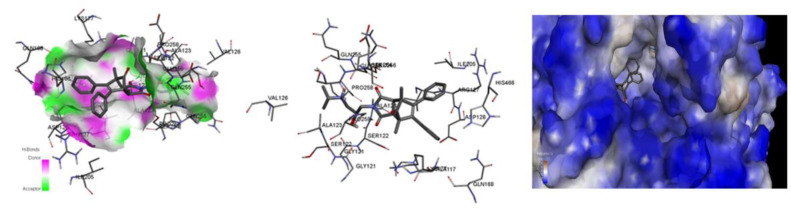
Bioactive conformation of compound III.

**Figure 7:**
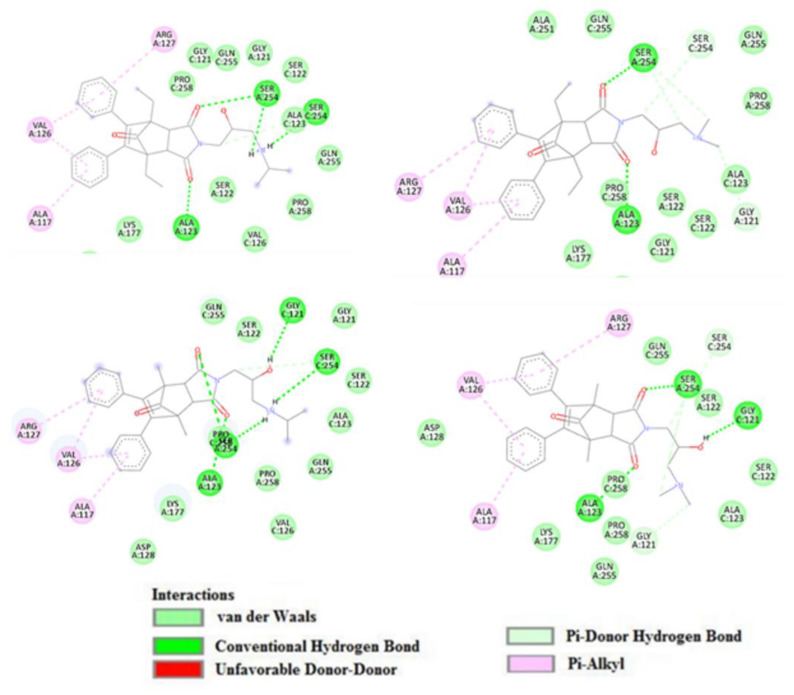
Predicted binding interaction between docked ligands (I, II, III, IV) and human catalase.

For each of the molecules, there was also present an intramolecular hydrogen bond, which additionally stabilized the potential bioactive conformation. While in the case of ethyl derivatives (compounds I and II) the bond was present between the OH group and the hydrogen of the amino group in the chain, in the case of methyl derivatives (compounds III and IV) the bond was between the OH group and the oxygen carbonyl of the imide group in the ring. Predicted binding interactions with the enzyme are depicted in Figure 6 and Figure 7.

## 3. Discussion

In this study, the inhibition potency (IC_50_) of aminoalkanol derivatives (I), (II), (III) and (IV) have been compared with 3-Amino-1,2,4-triazole as a specific catalase inhibitor [11] and other model compounds such as hydroxylamine, sodium azide, potassium cyanide [17]. A comparison was also made with other compounds, such as 3,3’-diaminobenzidine and its derivatives [18]. Comparing the inhibition potency (IC_50_) of aminoalkanol derivatives, for which the IC_50_ values are: (I) (IC_50_ = 11.40 mM), (II) (IC_50_ = 12.80 mM), (III) (IC_50_ = 10.30 mM) and (IV) (IC_50_ = 12.20 mM) with the value (IC_50_ = 6.00 mM) for 3-Amino,2,4-triazoles, it can be found that aminoalkanol derivatives are 1.72 to 2.03 times weaker CAT inhibitors in comparison to 3-Amino-1, 2,4-triazoles.

Comparing the inhibition potency (IC_50_) of (I), (II), (III) and (IV) aminoalkanol derivatives with inhibition potency of hydroxylamine, sodium azide, potassium cyanide [17], being the strongest catalase inhibitors, we can clearly see that the differences become even greater.

Inhibition potency of hydroxylamine (one of the strongest CAT inhibitors) is 0.02 mM, which in this case causes aminoalkanol derivatives to become 515 to 640 times weaker CAT inhibitors compared to hydroxylamine. The situation is very similar when we compare the IC_50_ values = 0.025 mM for sodium azide with IC_50_ values of 11.40 mM, 12.80 mM, 10.30 mM and 12.20 mM for (I), (II), (III), and (IV) aminoalkanol derivatives respectively. In this case, the aminoalkanol derivatives are 412 to 512 times weaker CAT inhibitors compared to sodium azide. In the case of potassium cyanide for which IC_50_ = 1 mM, aminoalkanol derivatives are 10.30 to 12.80 times weaker CAT inhibitors.

There are also nine 3,3’-diaminobenzidine derivatives with IC_50_ values ranging from 0.02 mM to 1 M. Comparing this time the inhibition potency of aminoalkanol derivatives with 3,3’-diaminobenzidine derivatives, it is clear that, in the case of eight of them, aminoalkanol derivatives are 515 to 12.80 times weaker CAT inhibitors. However, in one case (m-Phenylenediamine, IC_50_ = 1 M), aminoalkanol derivatives are 78.13 to 97.09 times stronger CAT inhibitors.

In this study, we also wanted to compare the values of the affinity strength (Ki) of aminoalkanol derivatives (I), (II), (III) and (IV) with the strength of the affinity of all CAT inhibitors listed above. Unfortunately, none of the authors provided information on the Ki value for the inhibitors they tested. The obtained Ki values for aminoalkanol derivatives (I), (II), (III) and (IV) of 1.61 mM, 3.04 mM, 1.32 mM and 1.79 mM, respectively, indicate the strongest affinity of compound (III) for CAT and the weakest affinity of compound (II) to this enzyme, which is consistent with all the kinetic parameters obtained from our research. However, due to the lack of data on Ki values from other studies, we were unable to perform a comparative affinity strength analysis of our inhibitors with those commonly considered as model inhibitors.

Our results showed that the inhibitors potency and affinity strength of all four derivatives depend on the type of four substituents (isopropylamine, dimethylamine, ethyl and methyl) present in the main chemical molecule of the compounds (I), (II), (III) and (IV) (Table 4). The strongest inhibitor of CAT turned out to be compound (III) with two methyl substituents and one isopropylamine in its structure. The weakest inhibitor was compound (II) containing two diethyl substituents and one dimethylamine (Table 4 and Table 5). The results of docking studies also show the convergence of the binding energies values of individual inhibitors with all kinetic parameters of the investigated catalase inhibition and thus additionally confirm the weak inhibitory strength of all four aminoalkanols derivatives.

## 4. Materials and Methods

### 4.1. Reagents and Chemicals

Catalase from human erythrocytes (EC 1.11.1.6, CAT), hydrogen peroxide (HPER), guaiacol (GCOL), sodium dihydrogen phosphate (NaH_2_PO_4_) and sodium hydroxide (NaOH) were obtained from Sigma–Aldrich (Poznań, Poland). Tetraguaiacol (TGCOL) was obtained from POCH (Gliwice, Poland). The compounds (I), (II), (III) and (IV) were synthesized and chemically analyzed (mass spectrometry and nuclear magnetic resonance spectroscopy) as described previously [29].

### 4.2. Instrumentation

A Beckman Coulter P/ACE MDQ CE system was used for electrophoretic analysis. The instrument had an autosampler along with a DAD detector. All CE parameters were controlled using Karat software (v. 32). Separation was carried out using eCAP fused-silica capillary (total length: 30 cm, effective length: 20 cm, inner diameter: 50 μm, outer diameter: 375 μm). The Autodock Vina program was used to calculate the docking parameters.

### 4.3. Capillary Electrophoresis (CE) Conditions

Electrophoretic separations of catalase (CAT), hydrogen peroxide, guaiacol (GCOL) tetra guaiacol (TGCOL), and inhibitors (I), (II), (III) and (IV) of catalase, by CE was performed using 100 mM (NaH_2_PO_4_) phosphate buffer as a background electrolyte (BGE) adjusted with 100 mM sodium hydroxide to the pH = 7.5. The samples were injected under 5 psi pressure for 3 s. The experiments were performed under constant current conditions (97 μA). The separation temperature was 25 °C, and the detection wavelengths were set at 214, 274 and 470 nm. Before the assay, the capillary was conditioned according to the previously described procedure [32]. 

Sample mixtures containing catalase (CAT), hydrogen peroxide (HPER), guaiacol (GCOL), tetra guaiacol (TGCOL), and inhibitors (I), (II), (III) and (IV) were introduced automatically by pressure injection (with 5 psi pressure for 3 s on the sample solution). At the day’s end, vials with anode and a cathode buffer were emptied and a running buffer was filled again at the start of the next day before the analysis. The detector wavelength was fixed at 470 nm. Hydrogen peroxide was not detected at wavelength of 470 nm. The appropriate parameters for analyzing CAT, TGCOL and (I), (II), (III) and (IV) inhibitors were as follows: phosphate buffer (pH = 7.5) of 100 mM concentration, 25 °C temperature, and 20 kV voltage.

### 4.4. Preparation of Stock and Working Standards

Primary stock standard solutions were prepared for compounds (I), (II), (III) and (IV) in deionized water (concentration of each = 100 mM). From this solution, successive solutions with concentrations of 2.5 mM, 5.0 mM, 10 mM, 15 mM, and 20 mM were prepared, which were used to determine kinetic parameters of catalase inhibition. Primary stock standard solutions were also prepared for GCOL and TGCOL at 100 mM starting concentrations. These solutions were then used to prepare further solutions with appropriate concentrations which were used to develop the linearity of the method. Standard HPER solutions of nine different concentrations (0.08, 0.16, 0.31, 0.63, 1.25, 2.50, 5.00, 7.50, and 10.00 mM) were prepared from a 100 mM hydrogen peroxide stock solution.

### 4.5. Sample Preparation

The CAT in the amount of 10 μg was dissolved in the 10 mL 100 mM phosphate buffer, pH 7.5 and divided into several portions, which were stored at −20 °C. One aliquot was thawed each day and the CAT activity was checked. For kinetic studies a catalase solution with a final concentration of 1 µg/mL was prepared. The working solutions of GCOL, TGCOL and HPER in appropriate concentrations were prepared immediately before use by dilution with the phosphate buffer according to the previously described procedure [32].

Catalase activity was determined according to the procedure described below (in accordance with the procedure described previously [32]). Briefly: In nine test tubes, 1.7 mL phosphate buffer (100 mM concentration, pH 7.5) containing CAT (1 μg/mL), 0.1 mL (400 mM) of (I), (II), (III) or (IV) inhibitor solution and 0.1 mL 400 mM guaiacol (GCOL) were added. To another tube (zero sample, tube 10), 1.8 mL of phosphate buffer (100 mM concentration, pH 7.5) containing CAT (1 μg/mL) and 0.1 mL 400 mM GCOL and 0.1 mL (400 mM) of (I), (II), (III) or (IV) inhibitor solution. The tubes containing solutions were preincubated for 2 min at 25 °C. Then, 0.1 mL HPER (at concentrations from 0.08 mM to 10.00 mM) was introduced into tubes 1–9, at an interval of 1 min. All 10 samples were incubated for 3 min at 25 °C. After incubation, the analytical samples were introduced into the capillary [32]. The color intensity of the obtained product was assessed at 470 nm. The results obtained from determining the inhibition type using the following equation are shown on the Lineweaver–Burk coordinate system in Figure 3 and Table 2:

$$\frac{1}{V}=\frac{Km}{Vmax*[S]}+\frac{1}{Vmax}$$

**Figure 8:**
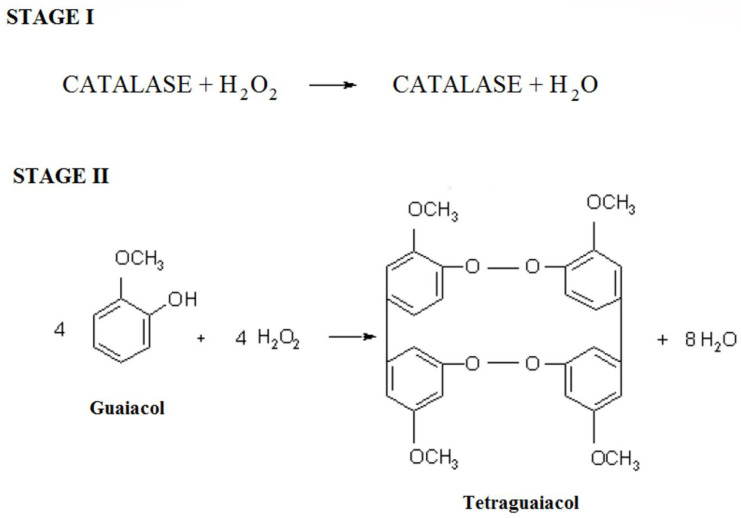
Reaction scheme for the determination of catalase activity.

Determination of inhibition effect of the compounds (I), (II) and (III), (IV) on CAT, was presented graphically as % activity depending on the changes in the concentrations of inhibitors (I) and (II). The IC_50_ values were obtained from activity (%) versus compounds (I), (II) and (III), (IV) plots. The inhibition constants (Ki) were calculated from the plotted Lineweaver–Burk curves for the four concentrations of the inhibitors (I), (II), (III) and (IV). The method was based on two steps (Figure 8).

Free hydrogen peroxide (stage 1), which has not been completely degraded by the enzyme by the action of inhibitors, reacts then with GCOL to form a specific amount of TGCOL (stage 2), the concentration of which depends on the CAT activity (Figure 8).

### 4.6. Docking Studies

To assess possible ligand-protein interaction, molecular docking experiments were performed using the PyRx docking tool via the Autodock VINA software [37,38]. The three-dimensional (3D) crystal structures of human erythrocyte catalase (PDB code: 1DGF) was obtained from the RCSB Protein Data Bank. The crystallographic structures were resolved with high resolution of 1.5 Å by an X-ray diffraction. The crystal structure of the protein was deposited in the PDB database in the form of a homotetramer and in each active center there was heme with a coordinated iron atom. The three-dimensional (3D) structure (PDB format) of catalase has been analyzed and prepared for docking in PyMOL software (DeLano Scientific LLC, San Carlos, CA, USA). Co-crystalised acetic acid molecules was removed from the structure and amino acids in proximity active site gorge was selected. The macromolecules were transferred to the PyRx tool, which automatically removes solvent particles, then adds hydrogen and performs Gasteiger charges calculations. The structural file has been uploaded to the PyRx utility linked to the Autodock VINA. The receptor and ligands was converted to the pdbqt format. The docking site was defined to include both the heme molecule as well as the amino acids defined as the binding pocket entrance area (ALA117, ASP18, GLN168, LYS177, VAL 182, TRP186, PHE200, HIS466). The grid box center was set to x = 29.9, y = 55.6, z = 72.3 and the dimension of grid box 25 × 25 × 30 was set to cover both sites in crystal structures. To increase the efficiency and accuracy of simulations exhaustiveness of docking was increased to 300. The generated docked complexes were selected based on binding affinity values (kcal/mol) and binding interaction patterns (hydrogen, hydrophobicity, and electrostatic), which were analyzed for higher scored conformation. Graphical representations of all docked complexes were made using the Discovery studio visualizer version 4.0 (BIOVIA, San Diego, CA, USA).

## 5. Conclusions

Catalase is one of the most important enzymes in our body whose task is to remove reactive oxygen species. Investigating the influence of new, potential therapeutic substances on the functioning of catalase is therefore of great importance. Aminoalkanol derivatives are promising substances with potential anti-cancer properties [29]. They are also (as shown in our previous studies) effective inhibitors of other enzymes that affect important disease entities [30,31,32]. However, the influence of aminoalkanol derivatives on the activity of catalase has not been studied so far. Therefore, taking the above into account, we conducted studies of their influence on the activity of this enzyme. 

Our study showed that CAT inhibition can be assessed simply with an in vitro drug metabolic system using CE analysis.

All kinetic parameters obtained thanks to our method such as Km, Vmax, slopes of Lineweaver–Burk, Ki and IC_50_ showed that all aminoalkanol derivatives are competitive CAT inhibitors, the inhibitory potency of which depends on the type of substituents present in the main structure of the molecule. Although the differences seem to be small, the developed CE method turned out to be so sensitive that it allowed to unequivocally determine the inhibitory strength of each of the four aminoalkanol derivatives. Based on the obtained results, it was found that derivative (III) (with two dimethyl and one isopropylamino substituents) was the most potent CAT inhibitor, while derivatives (I) (with two diethyl and one isopropylamino substituents) and (IV) (with two dimethyl substituents and one dimethylamine) turned out to be weaker CAT inhibitors. Derivative (II) (with two diethyl and one dimethylamine substituents) was in this case the weakest CAT inhibitor. The analysis of the docking studies of compounds (I), (II), (III) and (IV) showed that the investigated compounds cannot interact directly with the catalytic site of the enzyme due to the presence of a spatially expanded imide substituent, which additionally confirms their poor inhibitory strength against catalase.

The results of docking studies also show the convergence of the binding energies values of individual inhibitors with all kinetic parameters of the investigated catalase inhibition. Quite weak inhibitory strength of all four aminoalkanol derivatives causes a weak influence on the proper activity and functioning of catalase, which in this case is their big advantage.

## 6. Future Perspectives

Because the research results presented in this paper clearly show that all four aminoalkanol derivatives are very weak catalase inhibitors, it follows that they have very little influence on the proper functioning of one of the most important enzymes in our body. The weak inhibition of catalase by aminoalkanol derivatives, and thus the greater safety of their possible use in the treatment or regulation of other processes, prompts us to conduct further dynamic research on the possibility of using aminoalkanol derivatives for many other disease entities.

## Figures and Tables

**Table 1:** Regression equation and quantification for tetraguaiacol and guaiacol for six replicates for each sample (*n* = 6) in the concentration range 0.05–20.00 mM.

Linearity Range of Product (Tetraguaiacol) (mM)	R^2^	RSD (%)	LOD (mM)	LOQ (mM)	Regression Equation	Standard	Deviation
						Slope	Intercept
0.05–20.00	0.9999	2.38	0.01	0.04	y = 10.351 x − 1.232	±0.67	±0.058
**Linearity Range of Substrate 2** **(G uaiacol) (mM)**	**R^2^**	**RSD (%)**	**LOD (mM)**	**LOQ (mM)**	**Regression Equation**	**Standard**	**Deviation**
						**Slope**	**Intercept**
0.05–20.00	0.9999	3.14	0.02	0.07	y = 9.979 x + 1.695	±0.83	±0.067

LOD: limit of detection, LOQ: limit of quantification, RSD: relative standard deviation.

**Table 2:** Regression equation and quantification for hydrogen peroxide for six replicates for each sample (*n* = 6) in the concentration range 0.08–10.00 mM, in the presence of inhibitors (I), (II), (III) and (IV) at a concentration of 20.00 mM.

Concentration Inhibitors (I), (II), (III) and (IV) (mM)	Linearity Range of Substrate (Hydrogen Peroxide) (mM)	R^2^	RSD (%)	Regression Equation	Standard Deviation	
					Slope	Intercept
0	0.08–10.00	0.9989	2.74	y = 0.4308 x + 0.7933	±0.0047	±0.0036
(**I**) 20.00	0.08–10.00	0.9957	3.27	y = 0.5950 x + 0.8182	±0.0072	±0.0047
(**II**) 20.00	0.08–10.00	0.9979	3.01	y = 0.5109 x + 0.7981	±0.0053	±0.0042
(**III**) 20.00	0.08–10.00	0.9949	3.96	y = 0.6414 x + 0.8375	±0.0076	±0.0053
(**IV**) 20.00	0.08–10.00	0.9978	2.93	y = 0.5605 x + 0.7918	±0.0052	±0.0039

RSD: relative standard deviation.

**Table 3:** Determination of the inhibition type for CAT by comparing the Km and Vmax values between the control systems without inhibitor and the systems containing inhibitors (I) (II), (III) and (IV) at a concentration of 20.00 mM. An increase of the Km value with a constant Vmax value in comparison to the basic system indicates the competitive type of inhibition for all compounds ((I), (II), (III) and (IV)).

	Control System (Without Inhibitor)	System with Inhibitor (I)	System with Inhibitor (II)	System with Inhibitor (III)	System with Inhibitor (IV)
Km	**0.54**	0.73	0.64	0.77	0.71
Vmax	**1.26**	1.25	1.24	1.25	1.26
Type of inhibition		Competitive	Competitive	Competitive	Competitive

**Table 4:** The analytical data describing effects of catalase inhibition by aminoalkanol derivatives (I), (II), (III) and (IV) and regression equation for hydrogen peroxide for six replicates for each sample (*n* = 6) at the concentration range 0.08–10.00 mM, in the presence of inhibitors (I), (II), (III) and (IV), at a concentration of 2.50, 5.00, 10.00, 15.00 and 20.00 mM.

Compound (I)				
**Concentration (mM)**	**Straight Line Equation**	**R^2^**	**Slope**	
(I) 0.00	y = 0.4308 x + 0.7933	0.9997 ± 0.0008	23.31 ± 0.04	
(I) 2.50	y = 0.4604 x + 0.8103	0.9988 ± 0.0011	24.72 ± 0.05	
(I) 5.00	y = 0.4754 x + 0.8293	0.9995 ± 0.0009	25.43 ± 0.05	
(I) 10.00	y = 0.5167 x + 0.8259	0.9994 ± 0.0009	27.33 ± 0.06	
(I) 15.00	y = 0.54575 x + 0.8388	0.9996 ± 0.0009	28.62 ± 0.06	
(I) 20.00	y = 0.5950 x + 0.8192	0.9998 ± 0.0008	30.75 ± 0.04	
**Concentration (mM)**	**Km (mM)**	**Vmax (mM/min)**	**Ki (mM)**	**IC_50_ (mM)**
(I) 0.00	0.54 ± 0.03	1.26 ± 0.02		
(I) 2.50	0.56 ± 0.04	1.25 ± 0.04	11.73 ± 0.05	
(I) 5.00	0.57 ± 0.04	1.24 ± 0.07	9.76 ± 0.05	
(I) 10.00	0.62 ± 0.05	1.25 ± 0.05	3.57 ± 0.04	11.40 ± 0.05
(I) 15.00	0.65 ± 0.04	1.24 ± 0.07	2.74 ± 0.04	
(I) 20.00	0.73 ± 0.03	1.25 ± 0.05	1.61 ± 0.03	
**Compound (II)**				
**Concentration (mM)**	**Straight Line Equation**	**R^2^**	**Slope**	
(II) 0.00	y = 0.4308 x + 0.7933	0.9997 ± 0.0008	23.31 ± 0.04	
(II) 2.50	y = 0.4390 x + 0.7993	0.9992 ± 0.0015	23.70 ± 0.05	
(II) 5.00	y = 0.4503 x + 0.8151	0.9996 ± 0.0009	24.24 ± 0.05	
(II) 10.00	y = 0.4727 x + 0.7936	0.9996 ± 0.0010	25.30 ± 0.06	
(II) 15.00	y = 0.4874 x + 0.7985	0.9995 ± 0.0011	26.00 ± 0.04	
(II) 20.00	y = 0.5109 x + 0.7982	0.9998 ± 0.0008	27.06 ± 0.05	
**Concentration (mM)**	**Km (mM)**	**Vmax (mM/min)**	**Ki (mM)**	**IC_50_ (mM)**
(II) 0.00	0.54 ± 0.03	1.26 ± 0.02		
(II) 2.50	0.55 ± 0.05	1.24 ± 0.06	47.70 ± 0.08	
(II) 5.00	0.56 ± 0.05	1.24 ± 0.06	18.13 ± 0.06	
(II) 10.00	0.59 ± 0.04	1.26 ± 0.03	5.61 ± 0.05	12.80 ± 0.07
(II) 15.00	0.61 ± 0.03	1.25 ± 0.04	4.38 ± 0.05	
(II) 20.00	0.64 ± 0.03	1.24 ± 0.04	3.04 ± 0.03	
**Compound (III)**				
**Concentration (mM)**	**Straight Line Equation**	**R^2^**	**Slope**	
(III) 0.00	y = 0.4308 x + 0.7933	0.9997 ± 0.0008	23.31 ± 0.04	
(III) 2.50	y = 0.4682 x + 0.8124	0.9996 ± 0.0013	25.09 ± 0.04	
(III) 5.00	y = 0.4843 x + 0.8395	0.9998 ± 0.0006	25.84 ± 0.05	
(III) 10.00	y = 0.5452 x + 0.8396	0.9997 ± 0.0009	28.60 ± 0.04	
(III) 15.00	y = 0.5976 x + 0.8449	0.9997 ± 0.0010	29.58 ± 0.03	
(III) 20.00	y = 0.6414 x + 0.8375	0.9998 ± 0.0007	32.68 ± 0.02	
**Concentration (mM)**	**Km (mM)**	**Vmax (mM/min)**	**Ki (mM)**	**IC_50_ (mM)**
(III) 0.00	0.54 ± 0.03	1.26 ± 0.02		
(III) 2.50	0.57 ± 0.04	1.24 ± 0.07	8.86 ± 0.05	
(III) 5.00	0.58 ± 0.03	1.25 ± 0.08	8.71 ± 0.05	
(III) 10.00	0.65 ± 0.04	1.24 ± 0.07	2.77 ± 0.04	10.30 ± 0.03
(III) 15.00	0.71 ± 0.02	1.24 ± 0.09	1.80 ± 0.05	
(III) 20.00	0.77 ± 0.02	1.25 ± 0.02	1.32 ± 0.02	
**Compound (IV)**				
**Concentration (mM)**	**Straight Line Equation**	**R^2^**	**Slope**	
(IV) 0.00	y = 0.4308 x + 0.7933	0.9997 ± 0.0008	23.31 ± 0.04	
(IV) 2.50	y = 0.4522 x + 0.8021	0.9995 ± 0.0010	24.33 ± 0.06	
(IV) 5.00	y = 0.4662 x + 0.8167	0.9995 ± 0.0009	25.00 ± 0.05	
(IV) 10.00	y = 0.4930 x + 0.8052	0.9993 ± 0.0018	26.24 ± 0.04	
(IV) 15.00	y = 0.5162 x + 0.8146	0.9996 ± 0.0009	27.30 ± 0.05	
(IV) 20.00	y = 0.5605 x + 0.7918	0.9998 ± 0.0008	29.27 ± 0.03	
**Concentration (mM)**	**Km (mM)**	**Vmax (mM/min)**	**Ki (mM)**	**IC_50_ (mM)**
(IV) 0.00	0.54 ± 0.03	1.26 ± 0.02		
(IV) 2.50	0.56 ± 0.04	1.25 ± 0.03	14.23 ± 0.06	
(IV) 5.00	0.57 ± 0.05	1.24 ± 0.06	10.61 ± 0.04	
(IV) 10.00	0.61 ± 0.05	1.24 ± 0.06	4.26 ± 0.04	12.20 ± 0.06
(IV) 15.00	0.63 ± 0.03	1.23 ± 0.05	3.25 ± 0.04	
(IV) 20.00	0.71 ± 0.03	1.26 ± 0.02	1.79 ± 0.04	

**Table 5:** Effect of the type of substituents occurring in the main structure of aminoalkanol derivatives (I), (II), (III) and (IV) on inhibitory potency (IC_50_) and affinity strength (Ki) of CAT.

Compound	R_1_	R_2_	R_3_	Ki [mM]	IC_50_ [mM]	Docking Energy [kcal/mol]
Derivative (I)	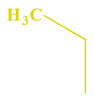	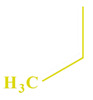	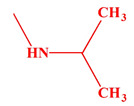	1.61	11.40	−8.6
Derivative (II)	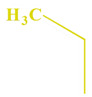	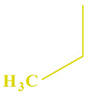	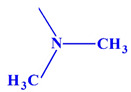	3.04	12.80	−8.4
Derivative (III)	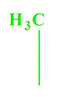	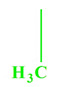	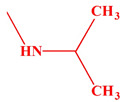	1.32	10.30	−8.9
Derivative (IV)	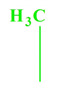	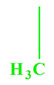	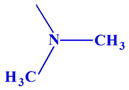	1.79	12.20	−8.2

## Data Availability

Not applicable.
